# Sharing is Caring: Minimizing the Disruption with Palliative Care

**DOI:** 10.7759/cureus.2321

**Published:** 2018-03-13

**Authors:** Abd Moain Abu Dabrh, Robert P Shannon, Richard J Presutti

**Affiliations:** 1 Department of Family Medicine, Mayo Clinic, Jacksonville, FL; 2 Department of Family Medicine/palliative Medicine Fellowship, Mayo Clinic Jacksonville, Fl

**Keywords:** shared decision making, minimally disruptive medicine, palliative and hospice care, cancer, coordinated care, primary care, specialty consultation, care models, caregiver, end of life care

## Abstract

There is an upward trend incidence of multiple chronic life-limiting conditions with a well-documented associated impact on patients and their caregivers. When patients approach the end of life, they are often faced with a challenging multidimensional burden while navigating a complex health care system. Patients and families/caregivers are faced with daily decisions, often with little or no frame of reference or medical knowledge. The “what, how, when, and where” puzzle during this challenging time can be overwhelming for patients and their families, and when clinicians do not contemplate this associated workload’s impact on patients and caregivers’ capacity for self-care, patients and caregivers scramble to find compensatory solutions, often putting their health care at lower priority. This consequently warrants the underlying importance of palliative care and integrating it into the patients’ health care plans earlier. There is increasing evidence from recent trials that supported implementing national policies regarding the early integration of palliative care and its role in improving the quality of life, increasing survival, and supporting patients’ and caregivers’ values when making decisions about their health care while possibly minimizing the burden of illness. The mission of palliative care is to assess, anticipate, and alleviate the challenges and suffering for patients and their caregivers by providing well-constructed approaches to disease-related physical treatments as well as psychological, financial, and spiritual aspects. Communication among all participants (the patient, family/caregivers, and all involved health care professionals) ought to be timely, thorough, and patient-centric.

Palliative medicine arguably represents an example of shared decision-making (SDM)—facilitating a patient-centered, informed decision-making through an empathic conversation that is supported by clinicians’ expertise and the best available evidence that takes patients values and preferences into consideration. Palliative care teams often consider the burden placed on patients and their caregivers, thus treatment plans would be assessed and introduced into the patients’ lives with reflection on the related workload and the potential capacity to take on those plans. Such an approach to pause-and-examine, understand-and-discuss, and assess-and-alleviate might provide a possible example of a health care system that is minimally disruptive to patients and their families. This is an opportunity to replace the information-filled encounter with a more constructive engagement and empowerment to all major stakeholders to participate—an axiom integral to palliative care. Using the best available evidence in caring for patients while enacting SDM, palliative care, primary care, and other subspecialty clinicians need to consider the significant workload and burden that comes with health care and thus explore pathways to minimize the disruption in patients and caregivers’ lives. As we collaborate to end cancer and all other mobdeities, we a need a concurrent movement to transform this disease-centered, payer-driven health care era to a rather patient-entered, thoughtful, and minimally disruptive one will benefit patients and physicians alike.

## Editorial

Hearing the words

“It seems that we are getting punished for living longer; but, I guess such is life.”

These were the words of a patient living with multiple chronic conditions for over two decades, now just diagnosed with advanced melanoma with metastasis to other organs. Regrettably, the patient had delayed seeking treatment fearing that the out-of-pocket payments would force the family to cancel their long-planned holiday gathering.

“Your health is more important than anything now,” I said.

“The memories we create as a family will outlast me; this is more important than anything else. I will eventually die, but those times will forever live in our minds and spirits,” the patient replied. It is within these sad but true words where opportunity lies, to work with patients to understand the options for care within the context of their lives, not just the context of their illness. This approach embodies the purpose of palliative care, the discipline of shared decision-making (SDM) within it, and its possible impact on the health care footprint for patients, their families, and their caregivers.

Understanding palliative care

The global and foreseen increase of cancer, dementia, and other chronic life-limiting conditions, and the associated impact on patients and their caregivers is well documented [1]. Regrettably, the upward trend continues in developed countries, thus underlying the importance of palliative care and integrating it into the patients' health care earlier. Palliative care clinicians assess, anticipate, and alleviate suffering by providing options and approaches to care for patients. The goal is to minimize disease burden and offer the best possible quality of life consistent with the patient’s goals. Palliative care “neither hastens nor postpones death” and supports the patient and family as the “unit of care” [2]; thus, a well-constructed plan of care addresses disease-related treatment alongside other physical, psychological, and spiritual aspects. Paramount to achieving these goals, communication among all participants (the patient, family, caregivers, and all involved health care professionals) ought to be timely, thorough, and patient-centric. This strategy advances disease-related treatment as well as other physical, psychological, and spiritual aspects, making this care truly patient-centric [2]. Palliative medicine arguably represents an example of shared decision-making (SDM); shared decision-making (SDM) focuses on facilitating a patient-centered informed decision-making through an empathic conversation rather than a jargon-filled clinical encounter [3]. This is accomplished by seeking to understand patients’ lives and honoring what matters to them in the context of their wishes, needs, cultural values, and spiritual beliefs. Palliative care physicians are well-suited to lead these types of conversations, where patients' goals and preferences are valued and incorporated in decision-making.

Highlighting the challenges

In the United States, Medicare reportedly spends about $170 billion on patients’ in the last six months of life. Despite the considerable spending, the demands of these patients are vastly unmet [2]. There are several barriers that hinder the goals to providing high quality of life to these patients.

Patients approaching the end of life endure multiple challenges dealing with complex medical morbidities and the consequences to their lives and to their families. Those affected must adjust and mobilize their capacity (i.e. ability) to respond to their health care workload (demands) [4]. The “what, how, when, and where” puzzle of navigating the health care system, insurance requirements, appointments, advance directives, and related tasks can be overwhelming. Furthermore, many patients and families may not understand what palliative care could offer during this challenging time. Patients may also encounter a fragmented health care setting that often leads to miscommunication between their primary and subspecialty clinicians. Additionally, patients and/or their families might hesitate or delay conversations about care options, struggling with the emotional challenge of relinquishing possible cure options and losing their family member. Another important factor to consider is the patients’ capacity to deal with their health and accompanying circumstance. When faced with the burden of medical treatment, patients themselves often struggle to find compensatory solutions. The result is often patients and/or their caregivers who minimize their needs or reshuffle life priorities with an end result that might sacrifice their health, compromise their quality of life, and/or result in blaming themselves or the system for not providing the proper care [4]. Think of a patient taking six different medications, undergoing thrice-weekly dialyses for four to six hours, and attempting to live independently. Contemplate the family who struggles to find the time to be present for their suffering loved ones while simultaneously attempting to care for their own family, to manage one or more jobs, and the fear of losing their job in the pursuit of that care. This is not an uncommon scenario—it is the norm that often compounds more distress and disruption in patients’ lives.

There are unique hurdles that palliative care clinicians face that may create barriers or minimize the value of palliative care. Primary and subspecialty care clinicians may not fully understand the role of palliative care and the need for its early integration in care plans, thus minimizing its demonstrated overall positive impact on the quality and quantity of life of the patients and caregivers [1, 2]. Involving palliative care teams may also be perceived by other clinicians as relinquishing aspects of patient care or may conflict with on-going treatment plans, creating a possible conflict in their patient-clinician relationship. As patients and/or their families might hesitate or delay palliative care conversations, clinicians may also hesitate to explore and follow up on these conversations, thus delaying involving palliative care and minimizing its potential impact in supporting better patient care and quality of life.

Using the evidence, sharing the decision, and minimizing the disruption

Though the value of palliative care is still understudied and is based mainly on observational studies, the impact of palliative care on patients’ lives is growing and health care policies are evolving to respond to these changes [1]. This mounting evidence from recent trials supports how early integration of palliative care improves the quality of life, increases survival, minimizes the burden of illness, and supports patients’ and caregivers’ values when making decisions about their health care [2, 5]. Within the context of SDM, in palliative care, it is recognized that patients and caregivers benefit from being involved in the decision-making early on and encouraged to express their wishes and values—think, feel, and speak. This occurs side-by-side as clinicians provide their expertise and are guided by the latest and best available evidence in determining their health care treatment plans.

Palliative care teams often consider the burden placed on patients and their caregivers. Treatment plans would be assessed and introduced into the patients’ lives with reflection on the workload related to it or the patients or their loved ones’ abilities to take on those plans. This occurs by offering a pause-and-examine approach over the consequences of recommended diagnostics or therapeutics. If a reform is due, arguably, palliative care may aim to deliver a minimally disruptive care [4].

For a long time, there was a lack of federal mandates that would require federally-funded programs and private institutions to report on the end-of-life quality measures and outcomes [2]. The recent policy changes implemented by the Centers for Medicare & Medicaid Services (CMS) provide palliative care options to patients without the risk of losing curative care. This is an optimistic paradigm shift at the policy-making level to explore ways, means, and models of care to increase access to crucial service, test new payment systems, and to respect the right of the patient to pursue options congruent with their wishes and goals (i.e. choosing to explore curative and palliative modalities simultaneously without being mutually exclusive).

Since the size and complexity of the health care system is growing, we as stewards of patient care, should support robust research that evaluates the capacity and workload of patient care and how better integration of palliative care could reduce the overall burden to the patient [1]. This could encompass striving harder to seek strategies that increase patient-caregivers’ capacity to endure their overwhelming tasks, as well as exploring pathways and methodologies that could curb unnecessary demands placed on the health care system. Focused research on the aspects of patient capacity and workload would also provide much-needed evidence to novel approaches to help current programs improve communication and the decision-making process with patients, their advocates, governing entities, and policy makers. For quality palliative care, attention to a minimally-disruptive, coordinated, and integrated care team could be the model of care to explore [1, 4]. The communication between care teams, the patient, and caregivers needs to commence at early stages and to engage all stakeholders and establish goals. These teams must aim to deliver expert-guided health care while engaging the patients and their families in a conversation that creates mutual discussion and dialogue, respectful of the patient’s goals. Additionally, primary or basic palliative care principles should be incorporated into the didactic and clinical training of medical trainees, primary care, and subspecialty clinicians to help them achieve early integration of palliative principles and appropriate referral to consultative palliative care specialists as warranted. This is highly important, especially in the reality of unmet needs for palliative and geriatric clinicians.

Moving forward

As we shoot for the moon to cure and stand up to cancer, Alzheimer’s, and all other chronically debilitating conditions around the world, we need a concurrent movement to transform this era from disease-centered, payer-driven health care to a patient-centric, thoughtful, and minimally disruptive one, and perhaps learn from what palliative care models offer. To palliate is to listen, to soothe, and to understand what our patients and their loved ones seek. We must work in synergy to provide the highest possible quality of care that fits the patient’s goal while respecting and dignifying their wishes and values, empowering shared decision-making, and minimizing any possible disruption due to the footprint of their health care. It is the least we can do and perhaps the best that we can do.
